# Hypoxia in Solid Tumors: How Low Oxygenation Impacts the “Six Rs” of Radiotherapy

**DOI:** 10.3389/fendo.2021.742215

**Published:** 2021-09-02

**Authors:** Andria Rakotomalala, Alexandre Escande, Alessandro Furlan, Samuel Meignan, Eric Lartigau

**Affiliations:** ^1^Oscar Lambret center, Tumorigenesis and Resistance to Treatment Unit, Lille, France; ^2^Univ. Lille, CNRS, Inserm, CHU Lille, UMR9020-U1277 - CANTHER – Cancer Heterogeneity Plasticity and Resistance to Therapies, Lille, France; ^3^Oscar Lambret Center, Academic Radiation Oncology Department, Lille, France; ^4^University of Lille, H. Warembourg School of Medicine, Lille, France; ^5^CRIStAL UMR CNRS 9189, University of Lille, Villeneuve-d’Ascq, France

**Keywords:** radiotherapy, hypoxia, radioresistance, HIF, thyroid cancer

## Abstract

Radiotherapy is an important component of cancer treatment, with approximately 50% of all cancer patients receiving radiation therapy during their course of illness. Nevertheless, solid tumors frequently exhibit hypoxic areas, which can hinder therapies efficacy, especially radiotherapy one. Indeed, hypoxia impacts the six parameters governing the radiotherapy response, called the « six Rs of radiation biology » (for Radiosensitivity, Repair, Repopulation, Redistribution, Reoxygenation, and Reactivation of anti-tumor immune response), by inducing pleiotropic cellular adaptions, such as cell metabolism rewiring, epigenetic landscape remodeling, and cell death weakening, with significant clinical repercussions. In this review, according to the six Rs, we detail how hypoxia, and associated mechanisms and pathways, impact the radiotherapy response of solid tumors and the resulting clinical implications. We finally illustrate it in hypoxic endocrine cancers through a focus on anaplastic thyroid carcinomas.

## Introduction

**Figure 1:**
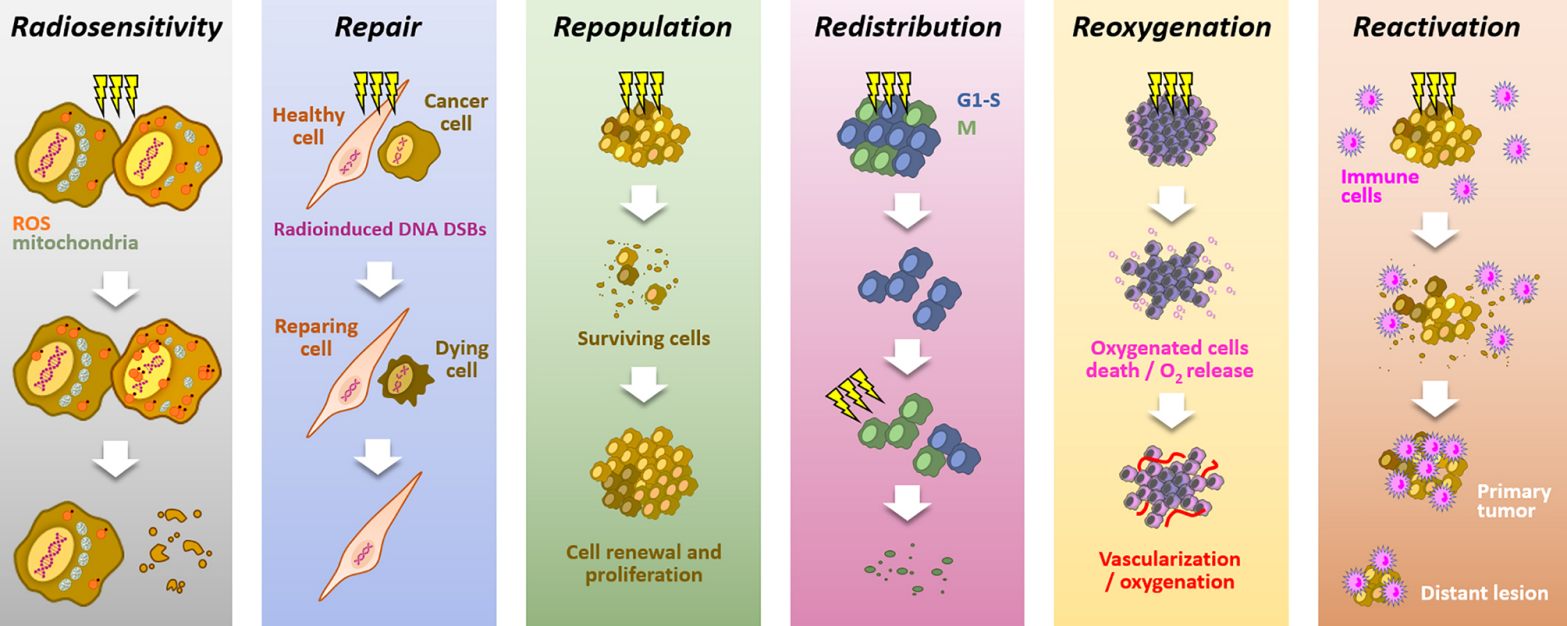
The six “Rs” dictating the response to radiotherapy. Radiotherapy response depends on six parameters: *Radiosensitivity*, refers to the cell-intrinsic mechanisms (*e.g.*, metabolic adaption, ROS detoxification), explaining differences in cell responses to irradiations; *Repair*, refers to the cell capacity to survive by repairing radio-induced damages (particularly DSBs), in theory, more characteristic of healthy cells; *Repopulation*, refers to the tumor cells capacity to grow following a radiotherapy fraction; *Redistribution*, refers to the progression of cancer cells from radioresistant cell cycle phases (*i.e.*, G1/S) toward more radiosensitive phases (*i.e.*, G2/M), between radiotherapy fractions; *Reoxygenation*, refers to the oxygen level recovery following irradiation, due to well-oxygenated cells death and tumor vascularization; *Reactivation*, refers to the triggering of a systemic anti-tumor immune response following irradiation-induced immunogenic cell deaths.

Because of its high cytotoxic potential in solid tumors, radiation therapy is a standard of care in many solid tumors (1, 2). The mechanism of action relies on ROS production, notably through water radiolysis, and DNA damages induction, especially double strand breaks (DSBs), leading to cell death. Aiming at killing cancer cells while preserving healthy cells, radiotherapy is mainly delivered through fractionated schemes. The success of fractionated radiotherapy depends on multiple sub-cellular, cellular, and microenvironmental parameters, together referred to as the “5Rs of radiation therapy”: *Repair* (of irradiation-induced DNA damages), *Redistribution* (of cells within the cell cycle), *Repopulation* (of tumor cells after radiation), *Reoxygenation* (of the surviving cells) and, more recently, intrinsic *Radiosensitivity* (3). Moreover, it is now accepted that immune response could play a critical role in radiotherapy response, leading to the emergence of a 6^th^ R: *Reactivation* (of the anti-tumor immune response) (4) (**Figure 1**).

However, numerous tumors fail to be controlled with radiotherapy, representing a significant cause of disease progression and mortality in cancer. There is a growing consensus that tumor heterogeneity is one of the principal explanations for treatment failure (5). In particular, oxygenation level is generally reduced and heterogenous within solid tumors, compared to the oxygenation found in associated healthy tissues (6, 7). In this sense, hypoxic regions are considered to be present in about 50% of solid tumors, and hypoxia is one of the most studied causes of radioresistance (8, 9). Indeed, hypoxia correlates with a poor prognosis after radiotherapy in various cancer types (10–12). Experimental evidence confirms the crucial impact of the low oxygen level (hypoxia) on the efficiency of cell irradiation.

**Figure 2:**
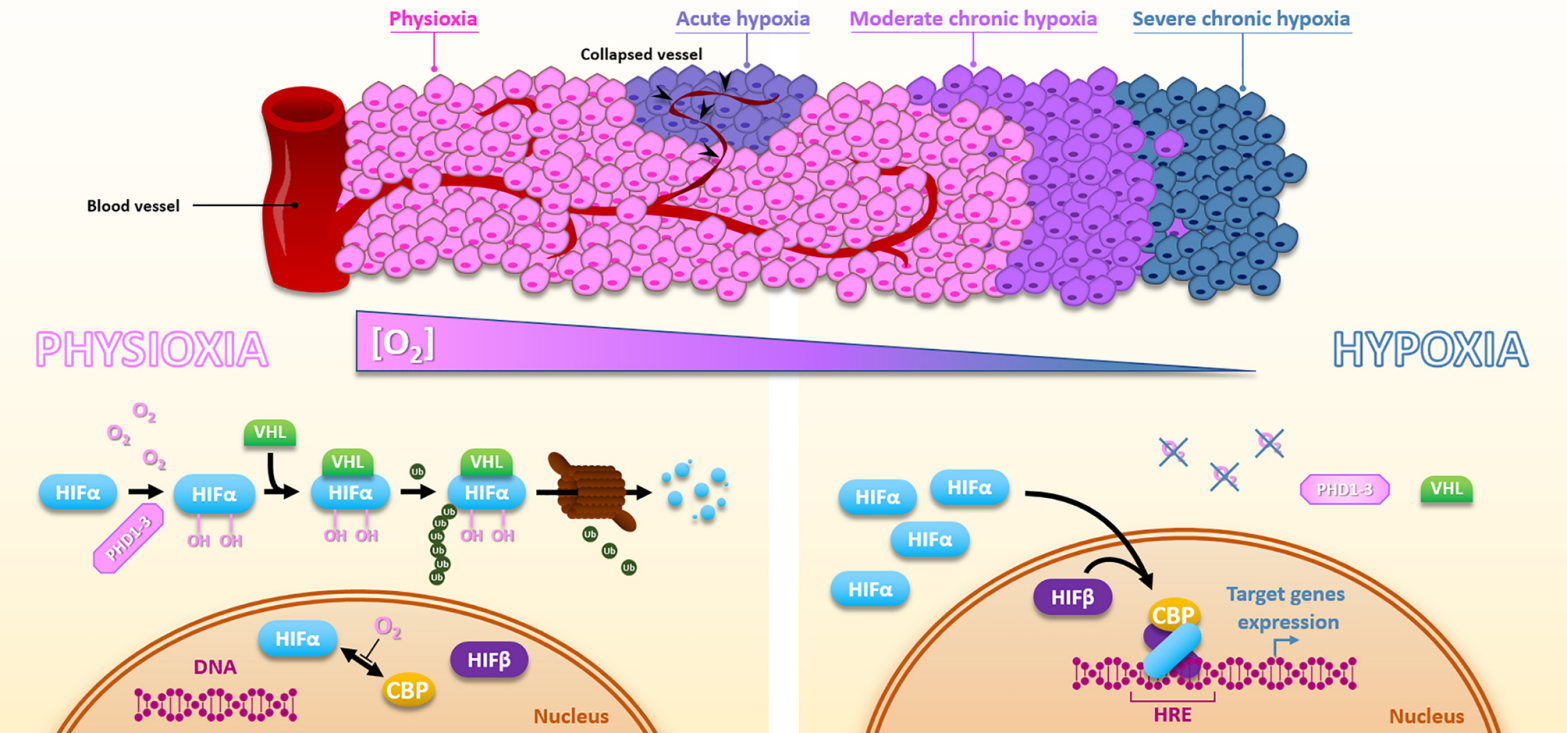
Hypoxia and HIFs regulation. Due to the distance to the blood vessels, cancer cells are well oxygenated or under moderate to severe chronic hypoxia. In addition, vessels collapse can cause sporadic acute hypoxic areas. Under physioxia, HIFα is hydroxylated by PHDs, poly-ubiquitinylated *via* VHL, and then degraded by the proteasomal way. Oxygen also inhibits HIFα-CBP interaction. Inversely, hypoxia inactivates PHDs, HIFα is subsequently stabilized, translocates into the nucleus, and forms a complex with HIFβ and CBP, which activates HREs-containing genes expression.

Hypoxia is a consequence of the high tumor cell proliferation rate and the abnormal structure of the tumor vasculature (13). Depending on the distance of the blood vessels and their transient collapses, hypoxia can be chronic, meaning diffusion-limited, or acute, meaning transient and perfusion-limited. Basically, within most solid tumors, oxygen level fluctuates between physioxia (about 8% O_2_, i.e., 60 mmHg), hypoxia (about 1% O_2_, i.e., 7.5 mmHg), and anoxia (0% oxygen) (14). The unquestionable master regulators of the transcriptional response to hypoxia are the HIF transcription factors (Hypoxia Inducible Factor), especially HIF-1. The regulation of HIF-1 is very dynamic to adapt quickly to the oxygen concentration. HIF-1 is composed of two subunits, HIF-1α and HIF-1β. Under physioxia, HIF-1α is hydroxylated on 402 and/or 564 proline residues by oxygen-dependent prolyl hydroxylase domain proteins (PHD1-3). This hydroxylated form will interact with the tumor suppressor protein Von Hippel-Lindau (pVHL), which recruits an E3-ubiquitine-ligase complex tagging HIF-1α by polyubiquitination for proteasomal degradation. Thus, in the presence of oxygen, the HIF-1α subunit is continuously produced and degraded, which regulates HIF-1 activity. Under hypoxia, oxygen-dependent PHDs activity is attenuated, HIF-1α is thus stabilized and accumulates to finally translocate to the nucleus where it dimerizes with the constitutively expressed HIF-1β subunit. Thus formed, the HIF-1 heterodimer will then recruit transcriptional coactivators (p300/CBP) and bind to hypoxia response elements (HREs) present in various gene promoters to initiate a transcriptional program leading to cell adaptation to hypoxia, notably through induction of metabolic switch, oxygen transport increase, and angiogenesis. In the same family, HIF-2 presents a similar structure. As HIF-1, HIF-2 is a heterodimer of the HIF-2α subunit combined with the HIF-1β one. The oxygen-dependent alpha-subunit HIF-2α is similarly regulated to HIF-1α (**Figure 2**). HIF-1 and HIF-2 modulate their own set of target genes and appear differentially regulated according to the hypoxia level and duration (15). By their role in hypoxia adaption, both HIF factors have a pleiotropic impact on the cell response to irradiation. Supporting this, in head and neck squamous cell carcinomas, HIF-1α and HIF-2α expressions positively correlate with a higher rate of local failure after chemoradiotherapy treatment (16, 17). Of course, hypoxia can also impact cellular behaviors through HIF-independent mechanisms and pathways. For example, severe hypoxia can induce endoplasmic reticulum stress and thus activate UPR (Unfolded Protein Response) pathways.

This review proposes to outline how low oxygen levels and hypoxia-associated tumor cell adaptions affect the six Rs of radiation therapy (here photon radiation therapy, the most widely used) and thereby its efficiency in solid tumors treatment.

## *Radiosensitivity*: How the Low Oxygen Level Intrinsically Primes Cancer Cells for Irradiation Resistance

“Radiosensitivity” defines the intrinsic sensitivity of tumor cells to radiation therapy. This property appears very heterogeneous within solid tumors and is impacted at two levels by hypoxia.

First, the molecular oxygen level directly impacts cell radiosensitivity *via* the “oxygen effect.” Actually, radiation-induced DNA damages are less likely to lead to cell killing under hypoxia (7). To explain that, the “oxygen fixation hypothesis” is based on the fact that oxygen reacts with radiation-induced DNA radicals to stabilize them. In a hypoxia or anoxia context, compounds containing sulfhydryl groups spontaneously reverse radiation-induced DNA radicals by reduction (18). *In vitro*, the “oxygen fixation hypothesis” is supported by the radioresistance induced by an oxygen depletion only during irradiation (19, 20). Another hypothesis formulated by Richardson and Harper suggests a central role for radiation impacts on mitochondria to explain the “oxygen effect.” In fact, radiations increase cell mitochondrial ROS content and disturb mitochondrial metabolism, which is more prejudicial for aerobic cells than hypoxic cells (21). The oxygen tension thus appears to be a determinant for the intrinsic tumor cell “Radiosensitivity,” and the “oxygen effect” may partly explain the radioresistant phenotype of hypoxic tumor areas.

**Figure 3:**
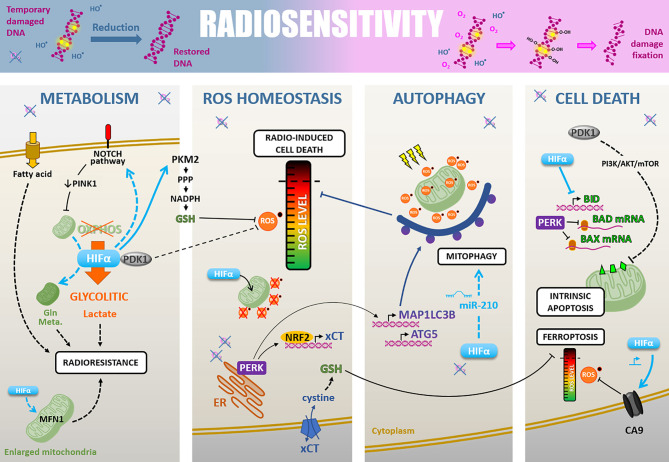
Radiosensitivity. Cell-intrinsic radiosensitivity can be linked to the fixation of radiation-induced DNA damages in the presence of oxygen, whereas a hypoxic environment favors the reduction of DNA radicals and return to a restored DNA (upper part). Hypoxia also impacts radiosensitivity through different cellular adaptions. i) The cell metabolism modifications, mainly PDK1-mediated glycolytic switch leading to lactate production, but also fatty acid uptake, NOTCH-dependent mitochondrial glutamine metabolism increase, and mitochondria fusion. ii) The ROS homeostasis is maintained under the critical level by PDK1 increase, HIFα-mediated GSH regeneration, HIFα translocation to mitochondria leading to their ROS production decrease, and PERK-mediated xCT expression promoting GSH synthesis. iii) Induction of HIFα- and PERK-mediated autophagy of ROS-productive damaged mitochondria. iiii) The cell death regulation through a HIFα-, PDK1- and PERK-mediated inhibition of antiapoptosis modulators BID, BAD, and BAX, and a HIFα-induced CA9 expression decreasing ROS level and inhibiting ferroptosis.

Besides, hypoxia promotes additional pleiotropic cellular adaptions through hypoxia-associated signaling pathways, which can prime cancer cells for radiotherapy resistance. These include cell metabolism rewiring, ROS detoxification, autophagy, and resistance to cell death (**Figure 3**).

### Cell Metabolism

Hypoxia modifies cancer cell metabolism (22). Yet, cell metabolism changes strongly correlate with radioresistance in various cancer models (23). The consensus is that hypoxia drives a metabolic switch from an OXPHOS metabolism toward a glycolytic metabolism (24). This glycolytic flux leads to an increased lactate production which correlates with a particularly poor prognosis in HNSCC and uterine cervix cancers treated by radiotherapy (25, 26). Moreover, the lactate concentration positively correlates with tumor hypoxia and relative resistance to a fractionated radiation therapy regimen (30 fractions within six weeks) in HNSCC xenograft models (27, 28). Following that, under hypoxic conditions, HIF-1α knock-down diminishes the tumor lactate level, increases basal and maximal oxygen consumption rate (OCR), and sensitizes HNSCC xenografts to high-dose single-fraction radiotherapy (29).

At the molecular level, various factors involved in hypoxia-induced metabolic modulations confer radioresistant properties to cancer cells. Among them, pyruvate dehydrogenase kinase 1 (PDK1), a HIF-1 target gene, inactivates the pyruvate dehydrogenase (PDH) responsible for pyruvate to acetyl-CoA conversion to supply tricarboxylic acid cycle (TCA) (30). So, PDK1 inhibits OXPHOS metabolism and forces cells to rely on glycolytic metabolism. In HNSCC, PDK1 expression appears to have a critical role in maintaining glycolytic metabolism under hypoxia through a HIF-1α dependent way and correlates with a dismal prognosis (31). Following the example of PDK1, another mechanism involved in the OXPHOS down-regulation and tumor aggressiveness under hypoxia (1% O_2_) is the modification of mitochondrial mass and morphology by PINK1 down-regulation through HIF-1-mediated NOTCH signaling activation in hepatocellular carcinoma cells, a cancer type highly resistant to radiotherapy (32).

Although hypoxia favors glycolytic metabolism over mitochondrial OXPHOS, mitochondria still keep a role in cell death and radiation therapy resistance under hypoxia (33). Chronic hypoxia (72h, 1% O_2_) actually modifies mitochondria morphology through a mitochondria fusion excess mediated by a HIF-1α, Mitofusin I, BNIP3, and BNIP3L dependent way in several cancer cell types (*i.e.*, colon, lung, cervix, renal cancer cells). These functional enlarged mitochondria confer a cell death resistance and could, by the way, prime hypoxic cancer cells to radioresistance (34, 35). Moreover, hypoxia can enhance mitochondrial glutamine metabolism of NSCLCC through a HIF-1α dependent manner and glutamine metabolism targeting can induce radiosensitization (36, 37).

Also, monounsaturated fatty acids are essential cellular compounds whose generation depends on oxygen. In lung adenocarcinoma cells, chronic severe hypoxia induces an adaptive up-regulation of SKG1 (human serum and glucocorticoid-inducible kinase) to promote unsaturated fatty acid uptake and thus shunt their oxygen-dependent generation. Matschke and colleagues showed that this hypoxia-linked adaption confers radioresistance *in vitro* in this model (38).

### ROS Homeostasis

Metabolism and ROS homeostasis are closely associated, and hypoxia significantly impacts ROS detoxification through metabolism rewiring and other ways. For example, PDK1, which expression increases under hypoxia, affects cell metabolism and thus maintains a low basal ROS level in hypoxic cells (30). As radiotherapy efficacy relies on ROS production, hypoxia can favor tumor cell-intrinsic radioresistance by promoting ROS detoxification processes.

In addition, to sustain a glycolytic metabolism, hypoxia can support glycolysis branched pathways such as pentose phosphate pathway (PPP), which participate in cellular redox homeostasis (39, 40). Mechanistically, hypoxia (1% O_2_) induces PKM2 (Pyruvate Kinase M2) isoform expression in breast cancer cells through HIF-1α recruitment on *PKM2* gene by KDM8 (41). The particularly weak activity of the PKM2 isoform slows the glycolytic flux down and favors glucose-6-phosphate accumulation to fuel PPP. During PPP, the 6-phosphogluconate dehydrogenase reduces NADP^+^ to NADPH which participates in reduced glutathione (GSH) regeneration, a cellular antioxidant highly involved in cellular ROS detoxification. So, by promoting PPP, hypoxia increases the cellular antioxidant potential and could counteract radiation therapy efficacy. Furthermore, severe cyclic hypoxia can increase GSH level through an up-regulation of two mitochondrial metabolite carriers, SLC25A1 and SLC25A10, in various cancer cell types (*i.e.*, glioblastoma, lung, and prostate cancer). These factors participate in NADPH cellular pool maintenance and sustain mitochondrial metabolism under hypoxia, notably fatty acids uptake, which has been linked to the radioresistance phenomenon (38). Consequently, severe cyclic hypoxia is associated with reducing basal mitochondrial ROS content and a high ROS detoxification potential, conferring resistance to radiotherapy (42, 43).

Beyond their canonical role of transcription factors, HIFs also protect cells from oxidative stress more directly. Indeed, under hypoxia or during oxidative stress, mitochondrial translocation of HIF-1α reduces mitochondrial ROS production and protects cancer cells from cell death (44), and possibly from irradiation-induced mitochondrial ROS production. Following the example of HIF-1α, HIF-2α can also prevent ROS overload and protect renal carcinoma cells from irradiation-induced cell killing (45).

In addition to the HIF-dependent response, severe hypoxia leads to endoplasmic reticulum (ER) stress and induces unfolded protein response (UPR). The PERK (PRKR-like ER kinase) axis of the UPR is particularly involved in cell survival during severe hypoxia or anoxia. In a subcutaneous murine glioma model, the tumor hypoxic fraction resists a high single dose of radiation therapy by activating the PERK/eIF2α/GADD34c axis, when HIF-1α is rather involved in post-irradiation re-progression. This PERK axis drives glutamate-cystine antiporter xCT expression, promoting GSH synthesis and cell radioresistance (46). In this sense, it has been shown that PERK phosphorylates the oxidative homeostasis master regulator NRF2 and promotes its nuclear translocation driving the expression of antioxidant factors, notably xCT antiporter (47–49). Küper and colleagues have recently shown that NRF2 stabilizes HIF-1α by direct interaction and plays a critical role in pancreatic and lung cancer cell radioresistance under hypoxia (1% O_2_) *in vitro* (50).

### Autophagy

In several cancer models, hypoxia exposure, as well as radiation treatment, lead to autophagy initiation (51). Autophagy preferentially occurs in hypoxic tumor regions and is observed along the entire gradient of hypoxia (52). Although autophagy can participate in radiation sensitivity in certain contexts, it is largely described as a cytoprotective mechanism, protecting the cell from extensive damages, notably after irradiation, by recycling damaged cellular components (*e.g.*, mitochondria). Thus, hypoxia-induced autophagy can constitute a radioresistance mechanism in several cancer models, as demonstrated in osteosarcoma cells, a highly hypoxic and radioresistant cancer type (53, 54). Moreover, targeting specific autophagy-related genes (*e.g., ATG3*) can sensitize pharyngeal, breast, and rectum resistant cancer cell lines to single-dose and fractionated irradiations *in vitro* (55).

At the molecular level, during severe hypoxia (<0.02% O_2_), the PERK axis of UPR sustains autophagy by inducing *MAP1LC3B* (microtubule-associated protein 1 light chain 3β) and *ATG5* (autophagy-related gene 5) expression *in vitro* and *in vivo*. This PERK-induced autophagy is a hypoxia-mediated adaption that diminishes cancer cell radiosensitivity, *in vitro* and *in vivo* (52). To a smaller extent, it has also been reported that the “hypoxamir” miR-210 can participate in the initiation of hypoxia-induced autophagy and promotes colon cancer cells radioresistance (56).

Finally, hypoxia-induced autophagy can highly reduce irradiation-linked oxidative stress and counteracts radiation therapy efficacy in various cancer cell models (*e.g.*, osteosarcoma and NSCLC cells) (53, 57).

### Cell Death Resistance

Radiation therapy relies on DNA damages and ROS overload induction to trigger cell death by various modalities (58). Among them, the cell death by mitotic catastrophe and the apoptosis processes are notably controlled by the BCL-2 family proteins, containing the pro-apoptotic members BID, BAD, and BAX (59). Yet, hypoxia (1-2% O_2_) and anoxia (<0.1% O_2_) correlate with the down-regulation of these proteins in colorectal cancer cells *in vitro*. In addition, these pro-apoptotic factors are widely absent from tumors hypoxic areas *in vivo*. Mechanistically, hypoxia-mediated *BID* down-regulation depends on its transcriptional repression by HIF-1α (60). Also, PDK1 (a HIF-1α target gene) drives PI3K/AKT/mTOR pathway activation, decreases BAX expression, and renders hepatocellular carcinoma cells resistant to irradiations (61). Likewise, the decrease of BAD and BAX protein levels can also be due to reducing their translation rate, possibly linked to the PERK activation observed under severe hypoxia (60). So, hypoxia can confer a radioresistant phenotype by down-regulate certain pro-apoptotic factors, which have been recently shown as essential for irradiation-induced apoptosis (58).

Besides apoptosis, recent studies suggest that other kinds of cell death are highly involved in radiation therapy efficacy. It’s notably the case for ferroptosis, an iron and ROS-dependent cell death (62), from which cancer cells are protected under hypoxia (63). Indeed, it has been shown that carbonic anhydrase 9 (CA9), a well-known target of HIFs factors, can participate in mesothelioma cells ferroptosis resistance by regulating ROS, lipid peroxidation, and mitochondrial Fe^2+^ levels under hypoxia (64). Also, by promoting ROS detoxification, notably through NRF2-mediated xCT expression (an anti-ferroptosis factor) and decreasing mitochondrial ROS production, hypoxia can counteract radiation-induced ferroptosis (65).

Since low oxygen level leads to selecting cells that can overcome and adapt to this stress, cyclic hypoxia promotes apoptosis-resistant cell expansion and further diminishes global radiation-induced cell death within the tumor. For example, cyclic hypoxia selects lung cancer cells resistant to the intrinsic apoptosis pathway. These selected cells harbor a radioresistant phenotype linked to a defective conformational change of BAX after irradiation (66). Also, a recent study defines hypoxia molecular hallmarks across 19 tumor types. It shows that hypoxic stress highly impacts the clonal evolution of tumors and drives an enrichment for specific molecular alterations (67). Among these hypoxic-enriched molecular alterations, TP53 loss of function is frequently observed and directly associated with an apoptotic potential loss. Of note, TP53 mutations drive the radioresistance phenomenon, notably in some pediatric high-grade gliomas characterized by their radioresistance (68).

To sum up, the low oxygenation both reduces radiotherapy efficacy *via* the physico-chemical “oxygen effect” and *via* hypoxia-induced biological adaptions. While hypoxia downplays the OXPHOS metabolism, the mitochondria are not inert and conserve a crucial role in hypoxia-mediated radioresistance phenomenon. The metabolic remodeling towards pathways known to enhance antioxidant protections, accompanied by increased autophagy, result in an exacerbate ability to maintain ROS homeostasis even after ionizing radiation. Last but not least, hypoxia drives and selects resistance to various radiation-induced cell death. Altogether, hypoxia undeniably enhances cancer cell’s intrinsic radioresistance and participates in radiotherapy treatment failure. In this frame, a phase I clinical trial (NCT01163487) evaluates tumor hypoxia by PET-scan in recurrent head and neck cancers and the possibility to use dichloroacetate (DCA), a drug targeting PDK1, in these tumors. Furthermore, a phase II clinical trial (NCT02432417) in glioblastoma assesses the efficiency of combining the standard chemoradiotherapy regimen with an autophagy inhibition by chloroquine. In this study, the hypoxia within the tumor is monitored by PET-scan imaging before and after the chloroquine treatment. A challenge for the effectiveness of autophagy-inhibition combined with radiotherapy remains to dichotomize contexts in which autophagy is cytoprotective rather than nonprotective, cytostatic, or cytotoxic.

## *Repair:* The Versatile Impact of Hypoxia on DNA Damage Repair

Radiation therapy causes direct and indirect (*via* ROS) DNA damages. The “*Repair*” defines the deferential capability of normal *versus* tumor cells to rely on DNA damage response (DDR) pathways after a radiotherapy fraction. Among radiation-induced DNA damages, double-strand breaks (DSB) are those responsible for the higher part of radiotherapy-induced cell death (69).

In eukaryotic cells, two major pathways drive DSBs repair; the non-homologous end joining (NHEJ) in most of the cases and the high-fidelity homologous recombination repair (HRR) (70). An increase in those DSBs repair mechanisms’ efficiency correlates with cancer cell resistance to radiation therapy (71, 72). Several studies have shown that hypoxia impacts those DSBs repair mechanisms (7, 73, 74). Nevertheless, hypoxia globally has a versatile role on cells’ DSBs repair capacity *in vitro*, either promoting or reducing it, depending on cellular model, hypoxia level, duration, and alternation with reoxygenation. For example, Kumareswaran and colleagues showed that continuous and very severe hypoxia (0.2% O_2_), as well as anoxia, cause a defective irradiation-induced DSBs repair in non-cancerous human fibroblasts (19). On the contrary, Wozny *et al*. recently showed that continuous but less pronounced hypoxia (1% O_2_) leads to an increased NHEJ initiation and correlates with radioresistance in HNSCC cells (75).

**Figure 4:**
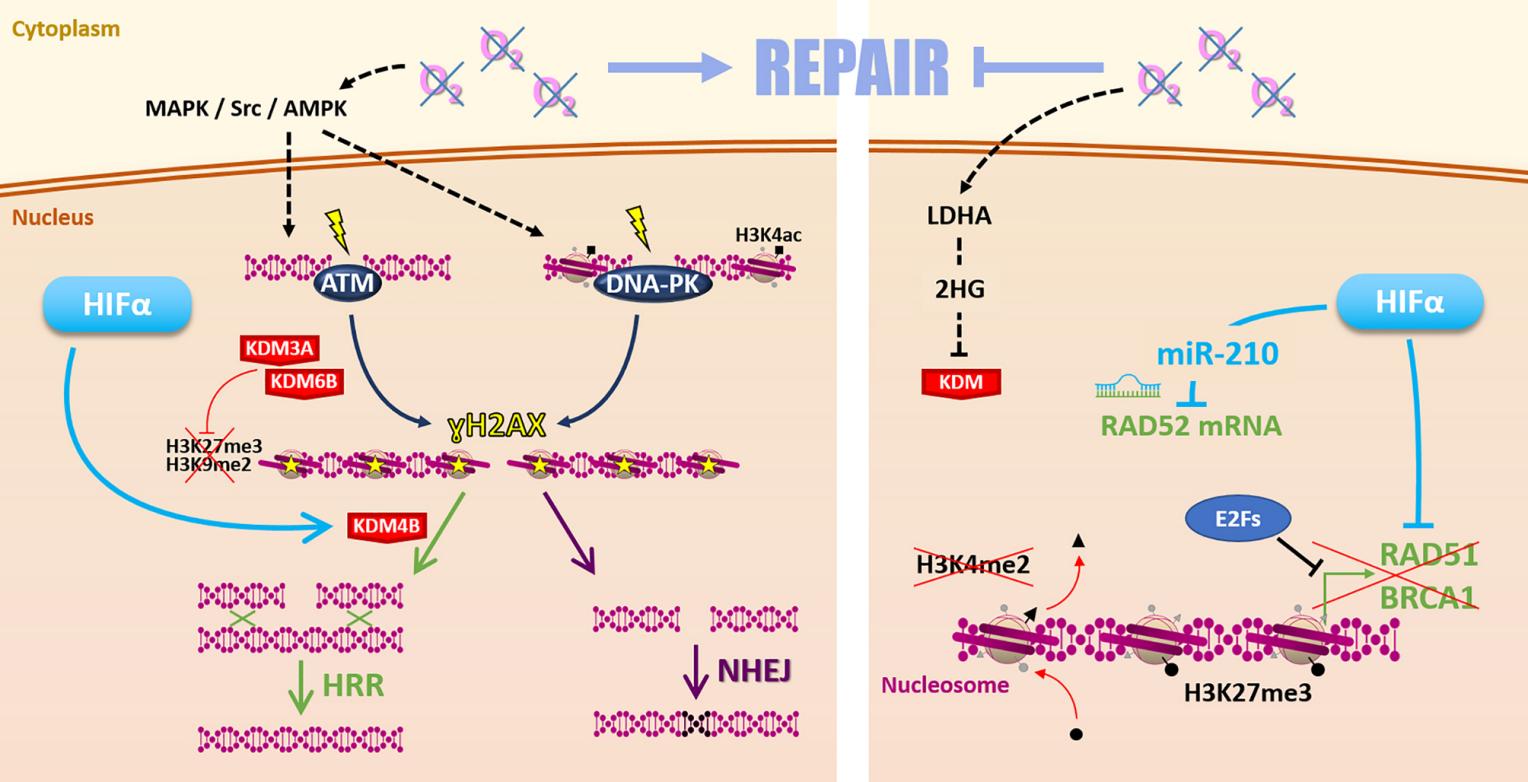
Repair. Hypoxia can favor radiation-induced DNA damages repair (left side) by MAPK/Src/AMPK-dependent ATM and DNA-PK activation. Hypoxia-induced modulation of KDM3A, KDM6B, and KDM4B expression changes the epigenetic landscape** **favoring DSBs repair by HRR or NHEJ. However, low oxygen levels can also affect the Repair (right side) notably *via* 2-hydroxyglutarate-dependent KDM inhibition. Moreover, HIFα and E2Fs inhibit HRR-related genes *RAD51*, *BRCA1* expression. Hypoxia-induced epigenetic changes (as loss of H3K4me2 and gain of H3K27me3 marks) inhibit these genes’ expression. HIFα can also inhibit *RAD52* mRNA through the miR-210.

Such discrepancies may find some explanations with various molecular mechanisms differentially impacted by hypoxia in different cellular contexts (**Figure 4**). On the one hand, it is frequently reported that hypoxia favors the initiation of DSBs repair mechanisms by increasing ATM (ataxia-telangiectasia mutated protein) and DNA-PKcs (DNA-dependent protein kinase catalytic subunit) expression and phosphorylation on ser1981 and ser2056, respectively. This notably occurs through MAPK, Scr, and AMPK signaling pathways (20, 76–78). Once activated, ATM and DNA-PKcs catalyze the phosphorylation of several substrates, notably histone H2AX at serine 139 (ɣH2AX), which signaled DSBs before effectors processing. Thus, hypoxia can prime cancer cells for DSBs repair initiation (79). In this sense, it has been recently demonstrated that hypoxia (1% O_2_) is associated with a higher ɣH2AX foci decay rate and enhanced RAD51 recruitment in irradiated HNSCC cells, suggesting a higher efficacy of HRR under hypoxia in this model (75, 80).

Contrarily, on the other hand, hypoxia reduces the expression of several key HRR effectors such as *NBN*, *MRE11*, *RAD51*, and *BRCA1* in different cancer cell models (20, 81, 82). Mechanistically, hypoxia-induced down-regulation of *RAD51* and *BRCA1* can be mediated by E2Fs factors (83, 84). Also, the reduced *BRCA1* and *RAD51* expressions in VHL-mutated cancers highlight the involvement of HIFs in HRR factors down-regulation and radiation-induced DSBs persistence (85). A mechanism of HRR genes down-regulation is driven by the HIFs-dependent miR-210, which targets *RAD52* mRNA (86). Altogether, the hypothesis that hypoxia down-regulates HRR to favor fast but error-prone NHEJ mechanism to repair radiation-induced DSBs emerged in some models (84, 87).

Concerning NHEJ pathway effectors, oxygen level impacts remain elusive and appear highly dependent on hypoxia conditions (7, 73). For example, in prostate and breast cancer cells maintained under severe (0.2% O_2_) and moderate (3% O_2_) hypoxia, respectively, both HRR-related genes (*e.g.*, *RAD51* and *BRCA1*) and NHEJ-related genes (*e.g.*, *Ku70*, *LIG4*, and *XRCC4*) are down-regulated (88, 89). On the contrary, in breast cancer, NSCLCC, and colon cancer cells, very severe hypoxia (0.01%O_2_) has no impact on NHEJ activity (82).

In addition, the deep relationship between hypoxia and epigenetic also impacts the “Repair” (90). Indeed, hypoxia-induced epigenetic modulations can directly impact the DDR orchestration, which relies on chromatin remodeling (91, 92). For example, hypoxia-enhanced acetylation at lysine 14 of histone 3 (H3K14ac) favors DNA-PK complex formation on DNA (79). This potentially primes hypoxic cells for post-irradiation DSBs repair initiation. It has also been shown that hypoxia can modify the epigenetic landscape (global loss of H3K27me3 and H3K9me2) by modulating epigenetic enzyme expressions, such as KDM3A and KDM6B and finally promotes DDR activity and radioresistance in HNSCC (80). Moreover, *via* HIFs, hypoxia can also increase KDM4B expression participating in HRR orchestration (93–95). Nevertheless, these KDM enzymes are α-ketoglutarate-dependent enzymes and are inhibited by L-2-hydroxyglutarate (2HG), a metabolite notably produced under hypoxia by lactate dehydrogenase A (LDHA) (96, 97). Thus, in that case, hypoxia-associated metabolism can also disturb HRR activity but by radiosensitizing cells (93). In this sense, hypoxia can also promote transcriptional silencing of the HRR-related genes *BRCA1* and *RAD51* through the induction of LSD1-mediated demethylation of the histone 3 lysine 4 (H3K4) and EZH2-mediated trimethylation of the histone 3 lysine 27 (H3K27) (98, 99).

Finally, hypoxia impacts “Repair” through various multimodal mechanisms leading to apparently antagonist cellular consequences. Globally, hypoxia appears to favor DSBs repair initiation and epigenetic orchestration but down-regulate HRR effectors. The consequent impact is highly dependent on the cancer model and hypoxia type. In this context, it seems complicated to develop a generalizable “Repair”-based therapeutic strategies to overcome hypoxic cells radioresistance. Still, a recent study demonstrated that, contrary to photon radiation therapy, hypoxia (1% O_2_) doesn’t enhance the repair of carbon ion irradiation-induced DSBs, suggesting that the choice of irradiation particles can overcome hypoxia-mediated repair enhancement in HNSCC (75). In this frame, a phase II clinical trial evaluates the benefit of an increased radiation dose and the use of carbon ion radiotherapy for hypoxic HNSCCs based on F-MISO-PET hypoxia-imaging (NCT03865277). Further studies in other cancer models and with different hypoxia parameters are needed to corroborate these promising findings.

## *Repopulation:* Hypoxia and Cancer Cells Renewal After Radiation Therapy

The *“Repopulation”* defines the renewal and proliferation of surviving cancer cells following irradiation. This phenomenon represents one of the main reasons for the failure of conventionally fractionated radiation therapy.

It is now widely accepted that cancer stem cells (CSCs) are involved in cancer progression and post-radiotherapy tumor recurrence through their self-renewal properties (100). Also, CSCs are frequently associated with hypoxic niches which favor stemness maintenance (101, 102). Hypoxia, particularly chronic hypoxia, promotes the self-renewal capacity of persistent CSCs (103). For example, hypoxia maintains CD133-positive glioblastoma cells in an undifferentiated state and enhances their self-renewal activity through a HIF-1α dependent way (104). In addition, in these cells, HIF-2α overexpression under hypoxia also plays an essential role in maintaining self-renewal capacities *in vitro* and *in vivo* (105).

Mechanistically, hypoxia favors CSC self-renewal by stemness-associated signaling activation. In breast cancer cells, HIF-2α stabilization under chronic hypoxia actives the WNT/β-catenin signaling pathway (106). Hypoxia can also induce AKT phosphorylation and thus participate in CSCs self-renewal by the AKT/WNT/β-catenin axis (107–109). Furthermore, the WNT/β-catenin signaling pathway drives post-radiotherapy progression in xenograft models of prostate cancer cells overexpressing HIF-1α (110). Another stemness-associated pathway potentially responsible for hypoxia-enhanced repopulation is the NOTCH pathway. NOTCH signaling pathway appears highly responsible for HIF-mediated maintenance of self-renewal properties in glioblastoma and breast cancer cells (106, 111). As WNT/β-catenin, the NOTCH signaling pathway is preferentially activated under chronic hypoxia in a HIF-2α dependent way (106), even if HIF-1α can also activate the NOTCH pathway by stabilizing NICD through direct interaction (111). STAT3 activation is a third pathway by which hypoxia promotes CSCs expansion. Through a HIF-1α-dependent way, hypoxia modulates glioma cells secretome, which induces STAT3 phosphorylation and subsequently promotes CSC self-renewal capacity *in vitro* and *in vivo* (112). Following these studies, HIFs factors appear highly involved in glioblastoma stem cells’ self-renewal properties that could explain the post-radiotherapy progression characteristic of this tumor type.

Hypoxia also plays a role in repopulation by regulating cyclin expressions, notably *via* HIFs factors, thus promoting radiation-surviving cancer cell proliferation. HIF-2α can drive cyclin D1 and cyclin D2 expression, notably through c-MYC activation for the latter, and promote cancer cell proliferation (113, 114). Following the example of HIF-2α, HIF-1α drives NOCTH1-mediated cell proliferation through cyclin D1 expression in melanoma cells under severe hypoxia (0.5%) (115). Also, hypoxia can impact cyclin expressions *via* the control of epigenetic enzymes. Indeed, Kim and collaborators have shown that, under hypoxia (1% O_2_), KDM4B induces cyclin A1 expression and favors gastric cancer cell proliferation following irradiation (95).

**Figure 5:**
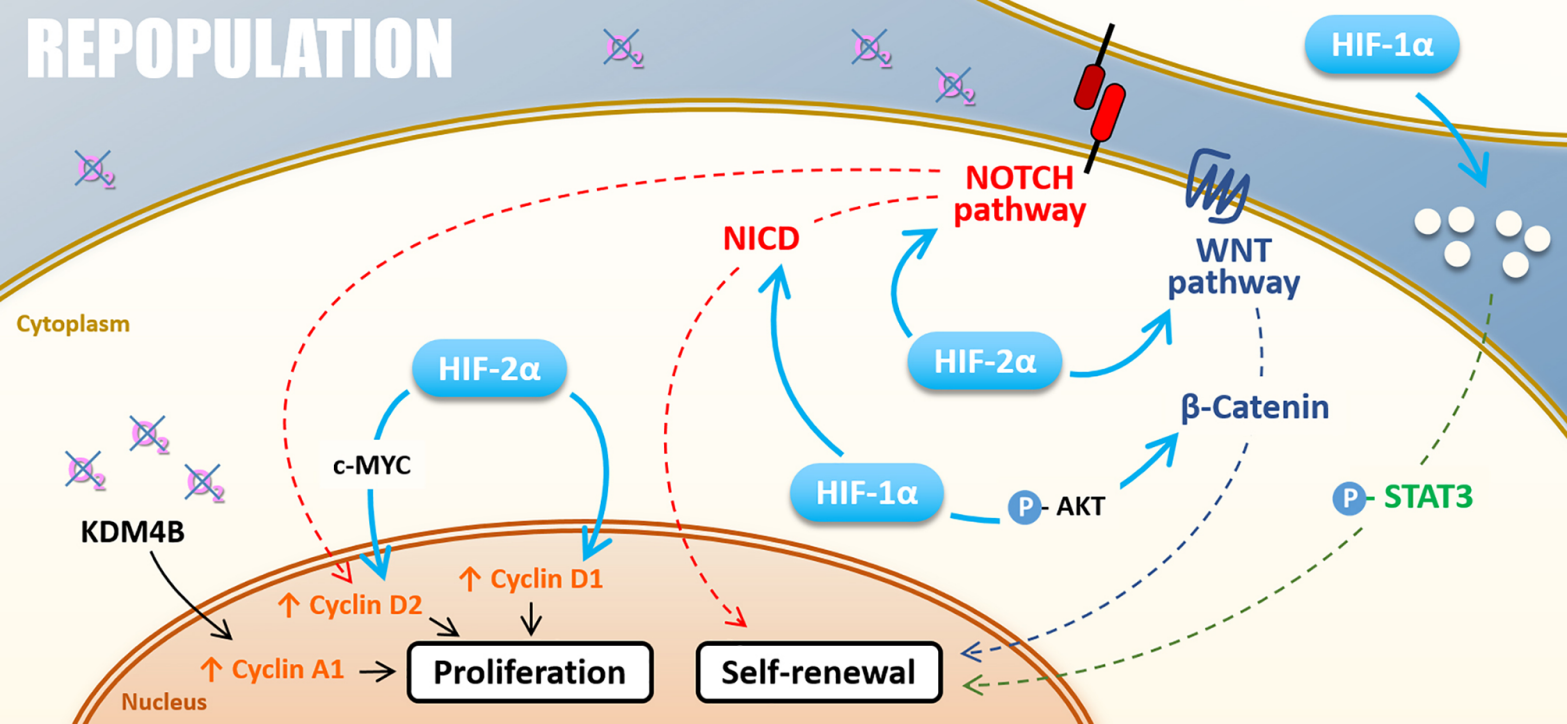
Repopulation. Under hypoxia, cell proliferation can be stimulated by KDM4B-dependant Cyclin A1 up-regulation and Cyclin D2 up-regulation driven by HIF-2α through c-MYC or thought NOTCH pathway under hypoxia. HIF-2α also directly induces Cyclin D1 expression. The self-renewal is positively regulated by NOTCH and WNT/β-catenin pathways activated by both HIF-1α and HIF-2α**. **HIF-1α also activates the STAT3 pathway and cell renewal through the modification of cell secretome.

To conclude, hypoxia widely impacts the “Repopulation” through HIFs master regulators (**Figure 5**). In several cancer models, chronic hypoxia and HIF-2α appear critical, either by favoring CSCs self-renewal *via* WNT and NOTCH signaling pathways or by modulating cyclin expressions through c-MYC activation for example. In this frame, the ɣ-secretase/NOTCH signaling pathway inhibitor RO4929097 has been tested in association with radiotherapy through several phase I/II clinical trials, notably for gliomas or breast cancers brain metastases (NCT01119599, NCT01217411). Nevertheless, these studies were prematurely terminated, and the clinical interest in inhibiting the NOTCH pathway in hypoxic tumors remains to be assessed.

## *Redistribution:* How Hypoxia Alters Cell Cycle Distribution Following Radiation Therapy

Cancer cells exhibit differential radiosensitivity depending on the cell cycle phase. Because cells in mitosis already pass through the last G2/M cell cycle checkpoint, they are the most sensitive to a mitotic catastrophe following radiations. Those in the G1 and S-phases are, on the contrary, more radioresistant (116). *“Redistribution”* defines the fact that these cells that survive the first fraction of radiation will progress into a more sensitive phase of the cell cycle and will be more sensitive to the following fraction. In this context, hypoxia, in particular acute hypoxia, *via* HIF-1α stabilization, counteracts radiation therapy efficiency by favoring cell-cycle arrest in less sensible phases (*i.e.*, G0/G1 and S-phases) (117).

Indeed, HIF-1α modulates many cell cycle regulation processes such as CHK1 (checkpoint kinase 1) activation and cyclin-dependent kinase inhibitor (CKI) expression like p21^CIP1^ (110). CHK1 and p21^CIP1^ are involved in cell cycle arrest in G1 or S-phase, by inhibiting CDC25A (cell division cycle 25A) activating-phosphatase or direct inhibition of the cyclin E/CDK2 complex, respectively. In this sense, in prostate cancer cells overexpressing HIF-1α, irradiation leads to a re-assortment in favor of G0/G1 and S-phase, in accordance with CHK1 phosphorylation and p21 up-regulation. This correlates with a radioresistant phenotype *in vitro* and *in vivo* (110). Mechanistically, under hypoxia (1% O_2_), HIF-1α displaces MYC from *CDKN1A* (*p21^CIP1^*) promoter and induces p21^CIP1^ up-regulation (118). Hypoxia also induces p27^KIP1^ and p57^KIP2^ up-regulation, two other CKIs targeting G1/S transition (119–121). In several cancer cell types (*i.e.*, colon, cervical and hepatocellular cancer cells), another hypoxia-induced mechanism that leads to G0/G1 and S-phase accumulation is the HIF-1α dependent down-regulation of CDC25A protein level (122). In addition to these mechanisms, it can also be assumed that hypoxia-induced ATM activation (described in the “Repair” section), which activates CHK2 and thus inhibits CDC25A, participates to cell cycle re-assortment in less radiosensitive phases during hypoxia (123).

In addition to their implication in “Repopulation” through their renewal properties, CSCs present in hypoxic niches can as well impact “Redistribution.” Indeed, CSCs can harbor a quiescent behavior under hypoxia. In glioblastoma, CSCs of the peri-necrotic zone, expressing HIF-1α, harbor a quiescent phenotype highlighted by the suppression of the RNA polymerase II phosphorylation at serine 2 (RNApII-S2P), a marker of transcriptionally less active quiescent cells (124). Moreover, in breast cancer cells, cyclic hypoxia selects slow-cycling CSCs, which accumulate in G0/G1 phase and are rarer in G2/M phases (125). At the molecular level, Ju and colleagues have recently shown that hypoxia (1% O_2_) induces *CSN8* (COP9 signalosome 8) expression, which correlates with several quiescence markers expression (*i.e.*, NR2F1, DEC2, and p27^KIP1^), a decrease of MYC expression, and a lower level of ki67 in colorectal cancer cells (126). Thus, CSN8 appears to be involved in a hypoxia-induced quiescent state and could, in this context, impact cell response to irradiation.

**Figure 6:**
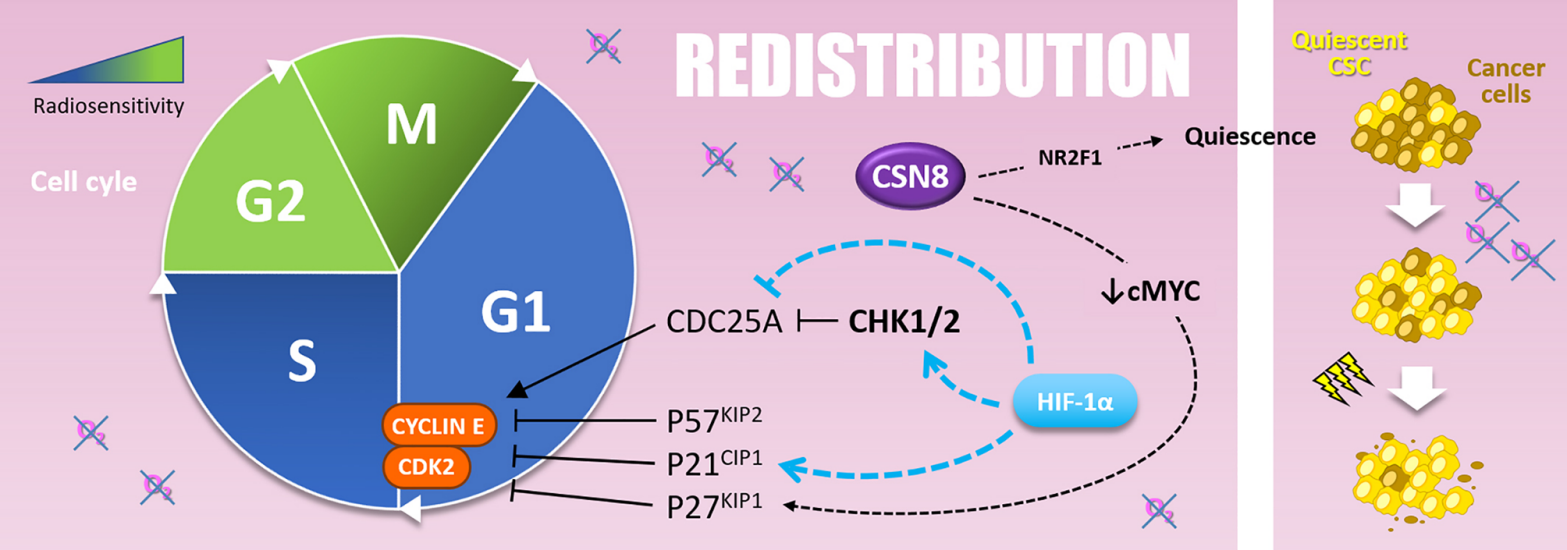
Redistribution. Cells are more resistant to radiotherapy in the G1 and, above all, in the S phase of the cell cycle. The G1-S transition is notably driven by the Cyclin E and CDK2, inhibited by P57^KIP2^, P21^CIP1^, and P27^KIP1^. Hypoxia upregulates P57^KIP2^ and P27^KIP1^ as well as P21^CIP1^ in a HIF-1α dependent manner. The overexpression of CSN8 correlates with cMYC decrease and** **P27^KIP1^ expression. It also correlates with NR2F1 quiescence marker expression. Quiescent CSC, selected by hypoxic stress, are more resistant to irradiation and participate in radioresistance (right side).

To sum up, hypoxia can counteract the “Redistribution” after radiation therapy by favoring cell cycle arrest within most radioresistant cell cycle phases (*i.e.*, G0/G1 and S-phase). Actually, HIF-1α promotes CKI expressions (such as p21^CIP1^) and CDC25A repression leading to cell cycle arrest in G0/G1 and S phases (**Figure 6**). In this sense, hypoxia can also maintain CSCs in a quiescent state. Nevertheless, determine in which context hypoxia counteracts the “Redistribution” (*via* cell cycle arrest) rather than favors the “Repopulation” (*via* cell expansion) after radiotherapy remains an open question.

## *Reoxygenation:* Radiation-Induced Hypoxia-Like Signaling Restricts Reoxygenation Effect

**Figure 7:**
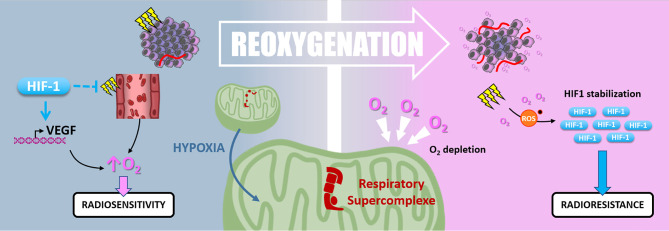
Reoxygenation. Radiotherapy induces tissue reoxygenation through O_2_ release and angiogenesis. Hypoxia also favors reoxygenation since HIF1 protects endothelial cells from irradiation and promotes VEGF expression. Nevertheless, hypoxia induces mitochondrial respiratory supercomplexes formation, which could persist after reoxygenation and cause high O_2_ consumption and depletion. Oxidative stress due to irradiation and reoxygenation stabilizes HIF-1, which can maintain radioresistance.

Hypoxic cells are up to three times more radioresistant than well-oxygenated ones. In this context, “*Reoxygenation*” defines the fact that, between radiotherapy fractions, well-oxygenated cells death leads to oxygen release, reduction of oxygen demand, and tumor bulk shrinkage allowing better oxygen diffusion and angiogenesis. Thus, the “*Reoxygenation”* turns back initially refractory hypoxic areas to a more radiosensitive state. Nevertheless, it is essential to note that irradiation and the subsequent reoxygenation induce oxidative stress that paradoxically stabilizes HIF-1 (127) (**Figure 7**).

At first, hypoxia appears to favor the tumor reoxygenation as *VEGF* is a target gene of HIF factors, and HIF-1 protects tumor endothelial cells from cell death following irradiation. According to these observations, hypoxia supports the oxygen level recovery inside the tumor and thus could enhance radiotherapy response subsequently.

Harada and colleagues have shown that hypoxic tumor areas are drastically reoxygenated 24 hours following irradiation. Surprisingly, this reoxygenation leads to an increase of HIF-1 activity through a glucose-dependent AKT/mTOR signaling activation (128). Moreover, Moeller and colleagues described that *in vivo*, HIF-1 pharmacological inhibition at the time of the reoxygenation post-irradiation sensitizes tumor cells (127). So, even after oxygen level recovering, HIF-1α-driven radioresistance mechanisms can be maintained. In addition, the high oxygen demand of some cancer cells can reduce the global oxygen availability inside the tumor and maintain a hypoxic environment. In this frame, in pancreatic ductal adenocarcinomas (PDAC) cells, an adaption mechanism to survive nearly anoxic conditions is the formation of mitochondrial respiratory supercomplexes. In this context, PDAC cells conserve oxidative metabolism and maintain their oxygen consumption despite a very low oxygen level (129). It can be assumed that this kind of cell, containing respiratory supercomplexes, disturbs radiotherapy-induced reoxygenation by maintaining oxygen depletion. Interestingly, in a recent study by Taylor and colleagues, the extent of tumor reoxygenation appears heterogeneous after five fractions of radiotherapy in murine orthotopic pancreatic PDX (patient-derived xenograft) containing hypoxic areas. This study showed that the level of reoxygenation dictates tumor growth inhibition following radiation therapy (130).

Finally, the precise role of “Reoxygenation” on radiotherapy response appears ambiguous. On the one hand, the consensus is that the oxygen level increase radiosensitizes tumor cells *via* the “oxygen effect”. Nevertheless, on the other hand, reoxygenation induces radioresistance through hypoxia-related signaling (*i.e.*, HIF-1 stabilization mediated by oxidative stress). Further studies are needed to determine whether initial tumor hypoxia affects the reoxygenation extent following radiotherapy, particularly fractionated protocols. Since the 60’s, the “Reoxygenation” concept gives rise to strategies aiming to increase tumor oxygenation during irradiation, such as using hyperbaric oxygen or administration of O_2_ at atmospheric pressure. Some of them were able to improve the local control of the tumor but appeared too difficult to set up in a clinical routine (131). More recently, several pre-clinical studies established a rationale for combining radiotherapy with HIF-1 inhibitor (132, 133), leading, for example, to a phase I clinical trial using PX-478 in advanced solid tumors (NCT00522652).

## *The 6^th^ R:* How Hypoxia Impacts “Reactivation” of Anti-Tumor Immune Response

**Figure 8:**
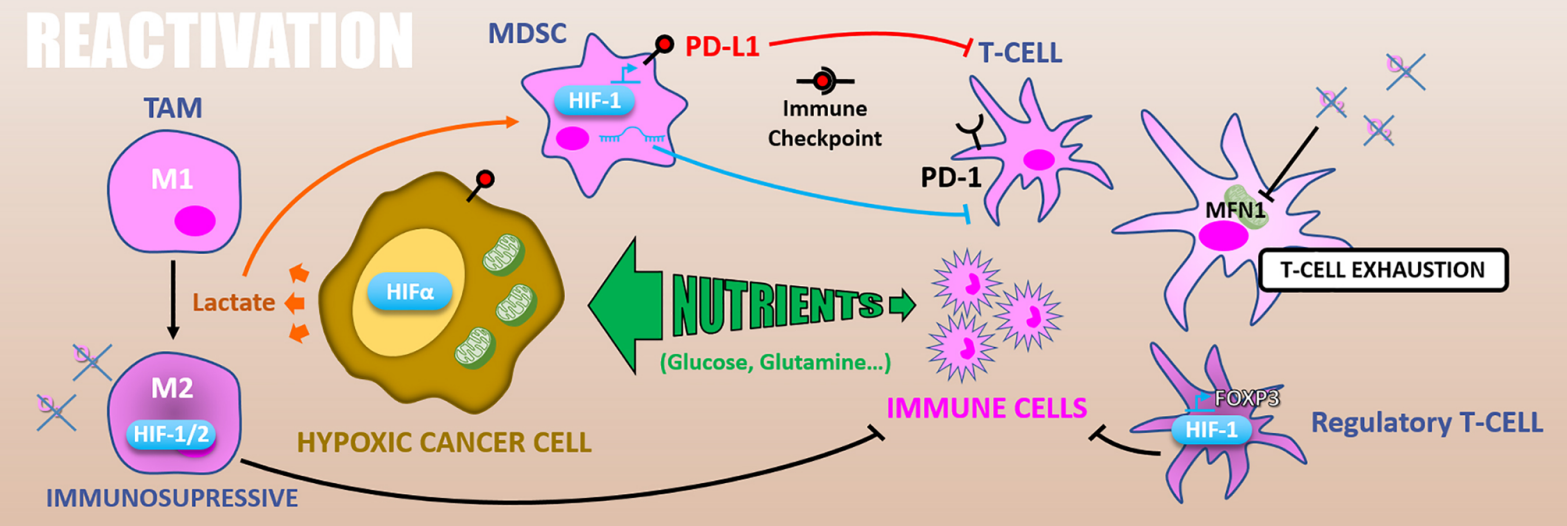
Reactivation. Because of exacerbated metabolism, hypoxic cancer cells consume nutrients from the microenvironment and starve immune cells. In addition, glycolytic metabolism produces lactate which polarizes TAMs in M2 immuno-suppressive and activates MDSCs inhibiting T-cells. HIFs expression in TAMs increases their immunosuppressive activity. Also, in MDSCs HIF-1 promotes PD-L1 expression that inhibits T-cell through PD-1/PD-L1 immune checkpoint. HIF-1α differentiates T-cells into immunosuppressive regulatory T-cells *via* FOXP3 expression. Finally, hypoxia can cause T-cells exhaustion by reducing their energetic metabolism through MFN1 down-regulation.

Radiotherapy has long been considered immunosuppressive through lymphocytes killing. Conversely, there is growing evidence assuming that irradiation efficacy may also rely on the induction of an anti-tumor immune response, depending on radiotherapy protocol, leading to a 6^th^ R rising: the “Reactivation” of anti-tumor immune response (4). Supporting this idea, ablative (high dose) radiation therapy efficacy on melanoma xenograft is significantly reduced in immunodeficient nude mice than in immunocompetent models. Indeed, ablative radiotherapy increases tumor antigens presentation by dendritic cells (DCs), stimulating effector T-cells priming and expansion (134). Furthermore, high single-dose irradiation triggers DCs, macrophages, and primed T-cells infiltration in the irradiated tumor (135). As mentioned above, ionizing radiations lead to various types of cell death, including immunogenic cell deaths (*e.g.*, necrosis, necroptosis), depending on the fraction dose. For example, a high dose, notably during ablative radiotherapy, can induce necrosis (136). Also, ionizing radiations can cause necroptosis, notably in endocrine cancers (*e.g.*, anaplastic thyroid and adrenocortical cancers) (137). Moreover, radiation-induced DNA damages leading to the mitotic catastrophe can end in necrosis or senescence. Then senescent cells could, in some cases, undergo necrosis (58). Altogether, these radiation-induced immunogenic cell deaths can trigger an anti-tumor immune response following radiation therapy (138). Mechanistically, immunogenic cancer cell deaths release tumor-associated antigens. These tumor-associated antigens are processed by antigen-presenting cells, which stimulates effector T-cells and triggers a systemic anti-tumor immune response. This is in line with a clinical observation called the “abscopal effect,” which consists of an immune-dependent regression of distant lesions after irradiation of the primary tumor (139, 140). So, innate and adaptive immune response players are involved in this radiotherapy-driven anti-tumor response. Yet, hypoxia modulates several immune player functions (141, 142) and participates in establishing an immunosuppressive microenvironment (**Figure 8**).

Besides, some nutrients (notably glucose and glutamine) essential for the anti-tumor function of immune cells (*e.g.*, T-cells, macrophages) are depleted in the tumor microenvironment because of consumption by cancer cells (143). This phenomenon by which cancer cells starve immune ones is called “metabolic competition” and appears amplified by hypoxia-related cancer cell metabolic remodeling.

In addition, glycolytic metabolism of hypoxic tumors results in lactate accumulation in the tumor microenvironment, leading to tumor-associated macrophages (TAMs) polarization toward an M2 immuno-suppressive phenotype (144) and can attenuate the radiotherapy-driven immune response. Moreover, the HIF-1α dependent lactate production by pancreatic ductal adenocarcinoma (PDAC) cells induces the activation of myeloid-derived suppressor cells (MDSCs) that reduces effector T-cells expansion and anti-tumor activity. This lactate-driven MDSCs activation is thus a major contributor to PDAC radioresistance (145). Supporting this work, the combination of hypoxia-inducible pro-drug with immune checkpoint inhibitors in prostate adenocarcinoma murine models highly reduces MDSCs density and functions inside the tumor. It thus restores T-cell infiltration and their anti-tumor activity (146).

Hypoxia also has a direct impact on the immune effector’s metabolism. As mentioned above, effector T-cells, notably T CD8^+^ lymphocytes, are required for optimal radiation therapy response. However, in nasopharyngeal carcinoma tissues, hypoxia causes T-cells exhaustion by reducing their energetic metabolism through an MFN1 (Mitofusin 1) down-regulation *via* a miR-24 dependent repression of the MYC transcription factor (147). HIF factors have as well an impact on immune effector cells. For example, HIF-1α stabilization and miR-210 expression are involved in hypoxia-enhanced MDSCs immunosuppressive functions (145, 148, 149). Also, in murine transgenic breast cancer models, it has been shown that TAMs exert an HIF-1α dependent immunosuppressive effect triggering a hypoxia-induced suppression of T-cell functions. Indeed, a specific knock-out of HIF-1α in macrophages decreases tumor aggressiveness (150). Similarly, HIF-2α suppression in TAMs reduces the tumor aggressiveness in a murine colon carcinoma model, indicating a HIF-2α role in hypoxia-induced immunosuppression (151). HIF-1α also favors CD4^+^ T-cells differentiation into immunosuppressive regulatory T-cells by promoting FOXP3 transcription factor expression (152).

Another way by which hypoxia can counteract immune reactivation following ionizing radiations is by increasing immune checkpoints proteins expression. In this sense, *PD-L1* (Programmed death 1 – ligand 1) is an HIF-1α target gene and is upregulated under hypoxia (0.1% O_2_) in MDSCs, in dendritic cells, as well as in cancer cells (153). Complementarily, hypoxia drives PD-1 (Programmed death 1) expression at the surface of T-cells and thus favors the PD-1/PD-L1 axis (147). Through CD86 upregulation in dendritic cells, hypoxia also promotes another well-known immune checkpoint, the CTLA4 (Cytotoxic T-lymphocyte-associated protein 4)/CD86 axis (154).

To sum up, radiation therapy induces immunogenic cancer cell deaths and can trigger a systemic anti-tumor immune response. However, hypoxia inside solid tumors counteracts this mechanism of action by promoting an immuno-suppression due to its impacts on cell metabolism and through HIFs factors, both in cancer and immune cells. As described in this section, hypoxia drives immune checkpoints proteins expression, encouraging the combination of radiotherapy with immune checkpoint inhibitors for patients with hypoxic tumors (155). Adding a hypoxia-inducible pro-drug to this treatment combination could durably restore anti-tumor immune response, and maximize radiotherapy response (146, 156).

## The Implication in Endocrine Cancers: Thyroid Carcinoma as an Example

As most solid tumors, endocrine cancers frequently exhibit hypoxic areas. Thyroid cancer is the most common endocrine malignancy. Anaplastic thyroid carcinomas (ATCs) account for 2-3% of thyroid gland neoplasms and are particularly aggressive tumors harboring a 3 to 4 months median survival (157). In this context, even though ATCs are often described as a radioresistant disease, adjuvant radiotherapy appears to increase the overall survival of patients with non-metastatic ATCs (158). Also, radiotherapy could offer a local control in advanced ATCs or for inoperable patients (159). Indeed, multimodal therapy with radiotherapy and chemotherapy (doxorubicin or cisplatin) is associated with the most extended survival and is considered a standard of care for these patients.

In ATCs, cancer cell metabolism relies on the reverse Warburg effect (*i.e.*, lactate uptake to fuel OXPHOS metabolism), frequently related to a hypoxic state (160, 161). In their study, Nakajo and collaborators concluded that glucose metabolism but also hypoxic conditions might be associated with progression in patients with metastatic thyroid cancer following radioactive iodine treatment (iodine-131) (162). Based on their metabolic profile, particularly their exacerbated glycolytic flux, a clinical trial assesses the benefit of using 18F-FDG-PET-guided radiotherapy in refractory thyroid cancers (NCT03191643). Most significantly, ATCs are necrotic tumors, supporting their hypoxic property (163). This hypoxic environment has been described to increase thyroid CSC-enriched side population, which are deeply involved in the “*Repopulation*” and also in the “*Redistribution*” parameters (164). Regarding “repopulation”, some authors have described a better overall survival depending on the schedule fractionation used, particularly in the case of twice-daily treatment (165). Furthermore, another modality, neutron radiation therapy, could also be explored as its effectiveness may be less sensitive to hypoxia (166). In the clinic, neutrons provides better survival for thyroid cancer in small patient series (167). However, these studies need to be confirmed, given the large discrepancies about this topic in the literature.While HIF-1α is not detectable in healthy thyroids, ATCs exhibit high levels of nuclear HIF-1α and consistently overexpress the HIF target gene CA9 (168). Of note, as previously mentioned in the “*Radiosensitivity*” section, the CA9 is involved in cellular ROS homeostasis and can participate in irradiation-induced ferroptosis resistance. Additionally, HIF-1α silencing has been described to promote thyroid cancer cells apoptosis (169). Thereafter, Kim and colleagues showed that pharmacological targeting of HIF-1α can be a promising approach for thyroid cancers treatment (170). Based on this *in vitro* rationale, further studies are still needed to assess if HIFs inhibition could improve radiotherapy efficacy and thus represent a promising approach in the clinic.

Altogether, there is currently a huge amount of evidence on the critical role of hypoxia in radioresistance, *via* a strong modulation of the 6Rs of radiotherapy. Preclinical and clinical studies are ongoing, which should now further help us in defining the potential of hypoxia-related strategies aiming to improve the efficacy of radiation therapy against cancers.

## Author Contributions

AR, AE, AF, SM, and EL wrote sections of the manuscript. All authors contributed to the article and approved the submitted version.

## Conflict of Interest

The authors declare that the research was conducted in the absence of any commercial or financial relationships that could be construed as a potential conflict of interest.

## Publisher’s Note

All claims expressed in this article are solely those of the authors and do not necessarily represent those of their affiliated organizations, or those of the publisher, the editors and the reviewers. Any product that may be evaluated in this article, or claim that may be made by its manufacturer, is not guaranteed or endorsed by the publisher.
